# Combined Pharyngeal Laceration and Laryngeal Fracture Secondary to Dog Bite: A Case Report

**DOI:** 10.7759/cureus.10828

**Published:** 2020-10-06

**Authors:** Samba Siva Bathula, Rebacca Mahoney, Aileen Kerns, Katrina Minutello, Noah Stern

**Affiliations:** 1 Otolaryngology, Detroit Medical Center, Michigan State University, Detroit, USA

**Keywords:** dog bites, animal bite, laryngeal fracture, pharyngeal laceration, neck injury, trauma, head and neck trauma

## Abstract

Dog bites are the most common animal bites, typically occurring in the head and neck region or extremities. The majority of dog bite-related injuries are superficial and require minimal medical intervention. Less commonly, dog bite injuries can be very serious when involving the airway, major blood vessels, or extensive tissue loss. To this day, there are very few case reports in the medical literature that describe severe dog bites and outline their management. We present a case of successfully treating an extensive pharyngeal laceration with a laryngeal cartilage fracture produced by an unvaccinated dog bite.

## Introduction

Dog bites are the leading cause of animal bites in the United States (US), with approximately 4.5 million events occurring each year [1]. On average, the annual incidence of emergency room (ED) visits due to dog bites is 1.1 per 1000 US population [2]. The majority of dog bite injuries occur in the head and neck region or extremities [3]. These are typically superficial requiring minimal medical intervention. Occasionally, dog bite injuries can be life threatening when involving the airway, major blood vessels, or extensive tissue loss [4]. Thus far, there is limited discussion on severe dog bite injuries and their management. We report a case of successfully treating an extensive pharyngeal laceration and cartilage fracture due to an unvaccinated dog bite.

## Case presentation

**Figure 1:**
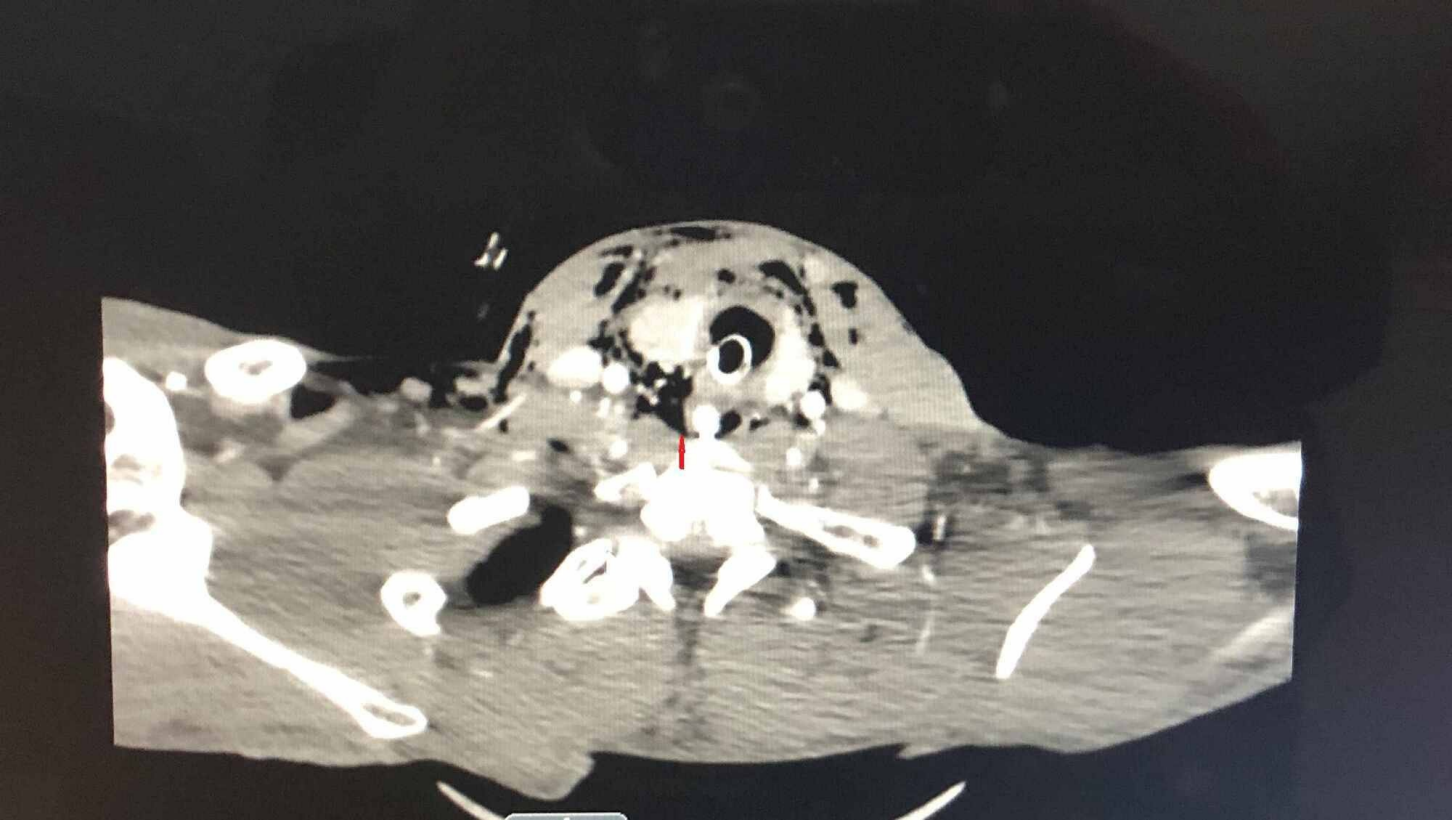
Right hypopharyngeal laceration-superior cornu of the thyroid cartilage A right neck laceration into hypopharynx through superior cornu of the thyroid cartilage with subcutaneous free air present. Arrow shows nasogastric tube in hypopharynx and free communication of hypopharyngeal air to neck subcutaneous air.

**Figure 2:**
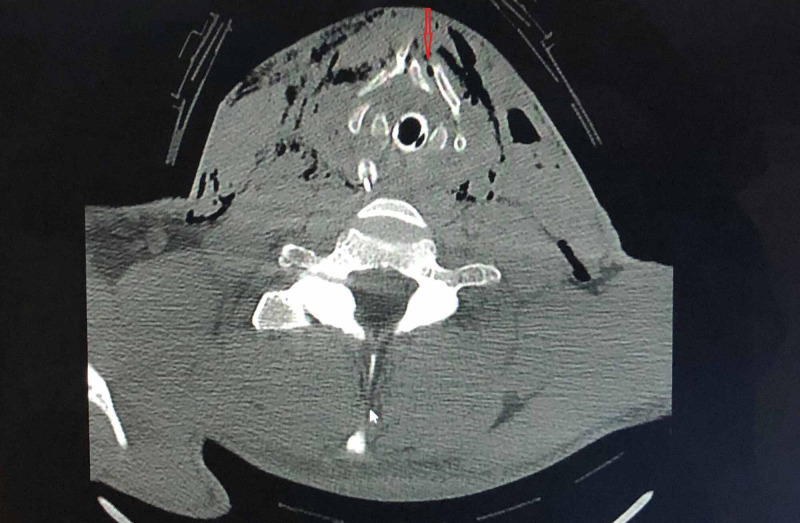
Thyroid cartilage fracture Multiple thyroid cartilage comminuted fractures at the laryngeal prominence are shown in arrow.

A 40-year-old male patient was brought to the hospital emergency room in an unconscious state with multiple neck wounds due to a bite from an unvaccinated Pitbull dog. He was immediately intubated by the anesthesia team and a computed tomography (CT) of the head, neck, chest, and abdomen was performed. CT scan showed a right neck laceration into the hypopharynx through superior cornu of the thyroid cartilage with subcutaneous free air (Figure 1). It also showed multiple thyroid cartilage comminuted fractures at the laryngeal prominence without any injury to the carotid artery and internal jugular vein (Figure 2).

**Figure 3:**
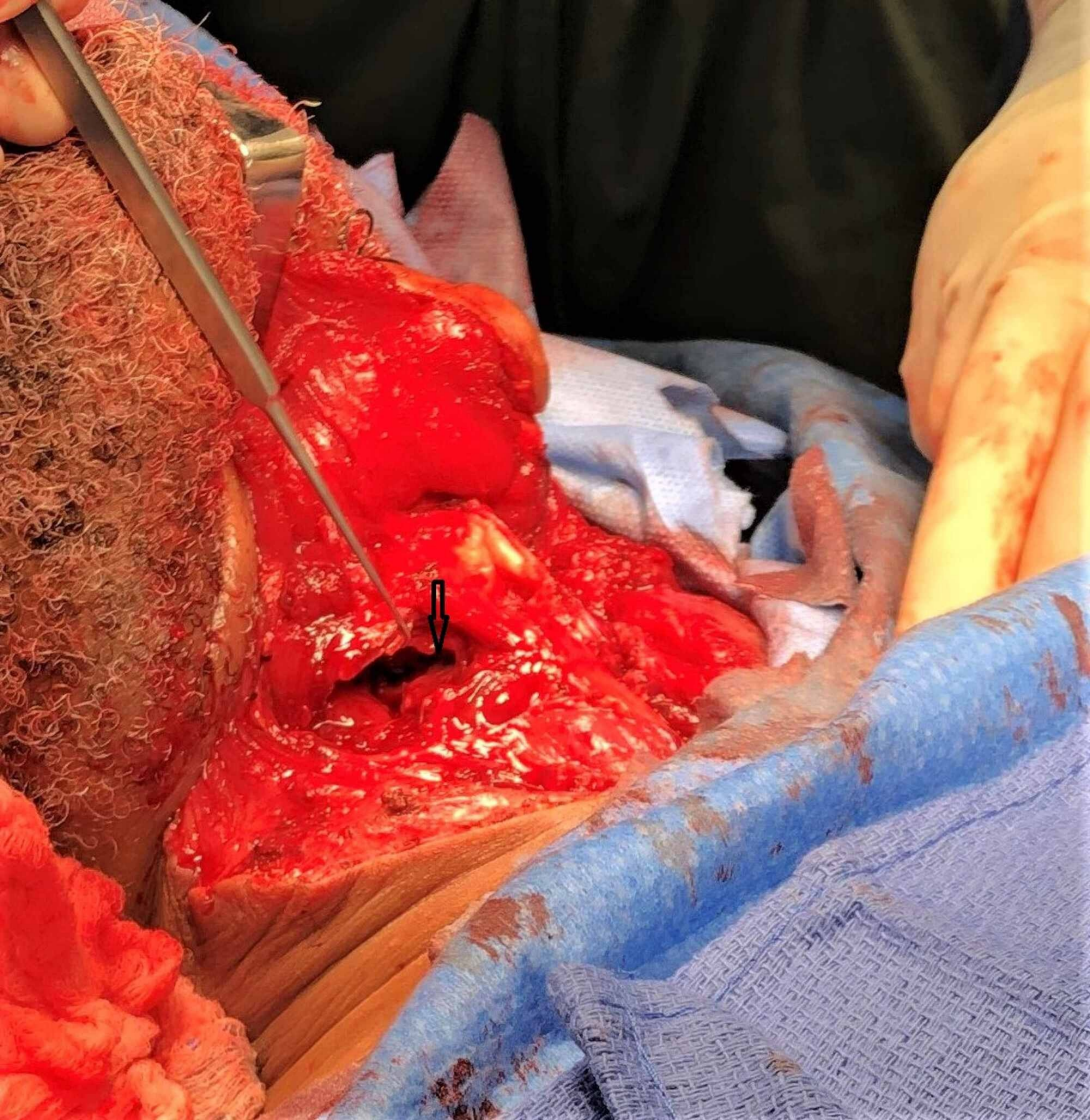
Right hypopharyngeal laceration A right laryngopharyngeal laceration at the level of greater cornu of the thyroid was able to be with the endotracheal tube in place.

**Figure 4:**
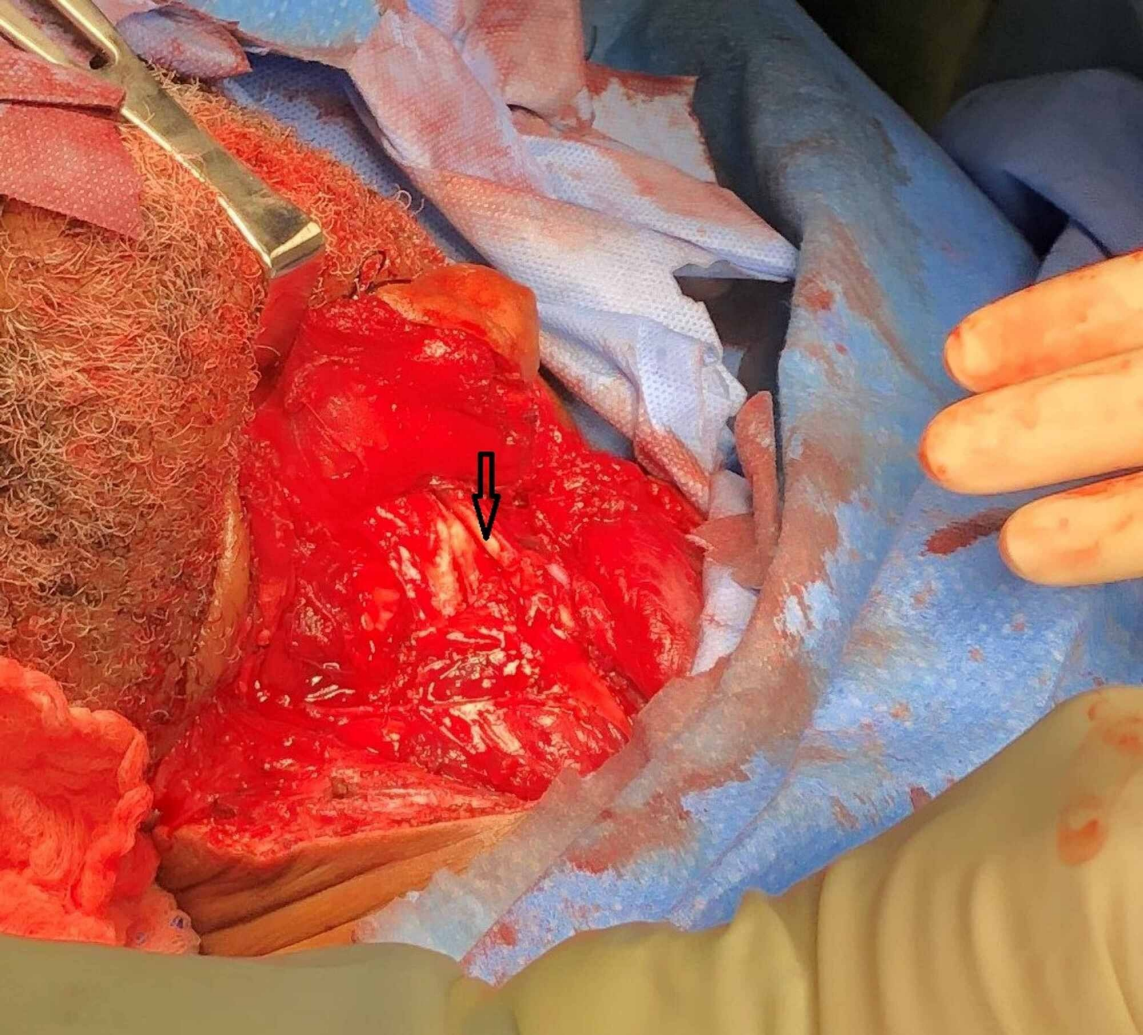
A thyroid cartilage laryngeal prominence fracture Anterior thyroid cartilage comminuted fracture.

The patient was taken to the operating room for a neck exploration. He was found to have a 3.0 x 1.0-cm right laryngopharyngeal laceration at the level of the superior cornu of the thyroid cartilage with active saliva freely flowing over the carotid sheath (Figure 3) and a 3.0 x 1.0-cm anterior thyroid cartilage fracture (Figure 4). The fractured cartilage was barely attached to the perichondrium, qualifying as a Group 4 laryngeal injury according to Schaefer Classification System. A laceration of the left ear and multiple skin lacerations in the anterior neck were also identified. No esophageal and tracheobronchial injuries were noted by esophagoscopy and bronchoscopy, respectively.

**Figure 5:**
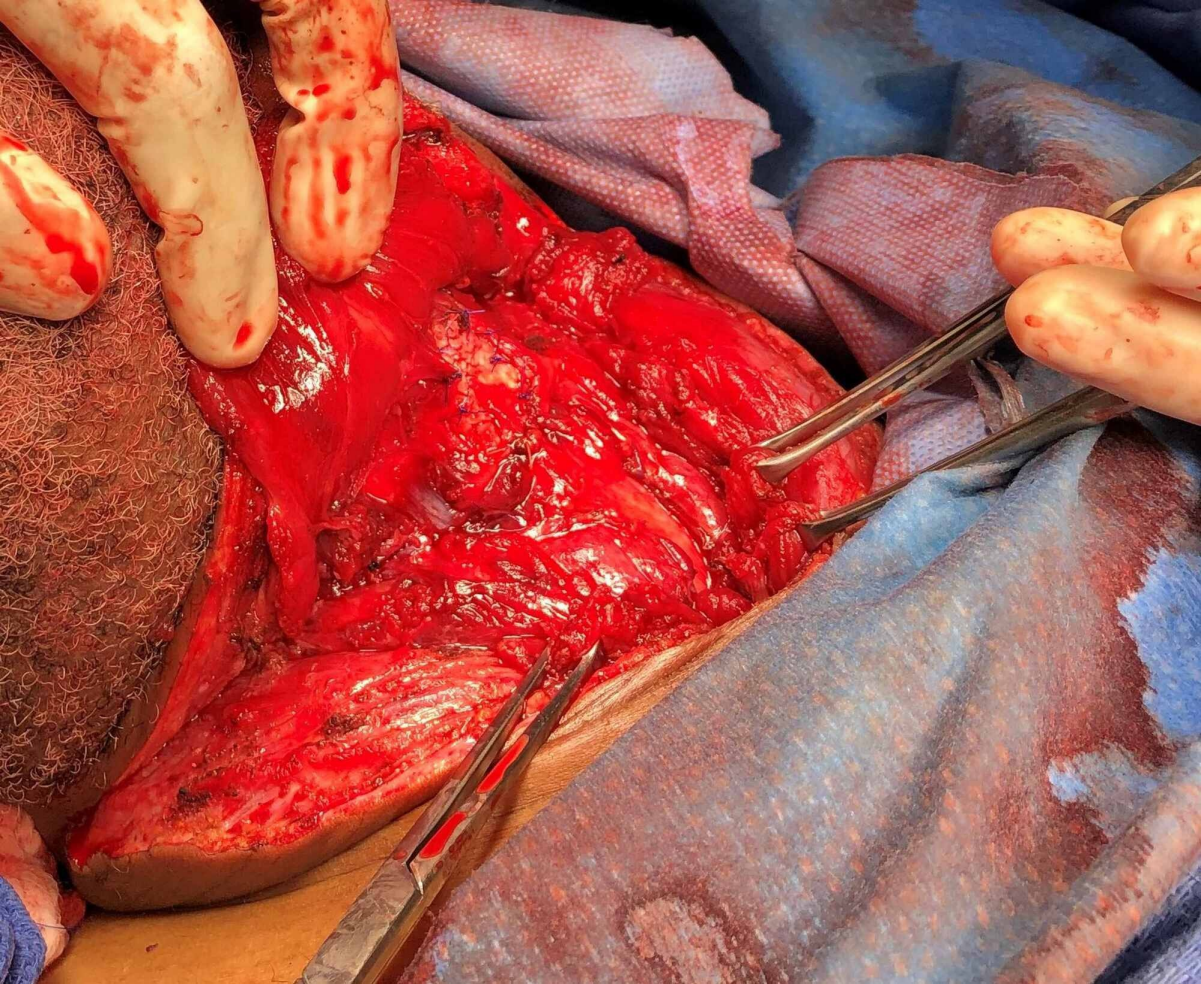
Anterior neck view after hypopharyngeal laceration and thyroid cartilage laceration repair The laryngeal anatomy after repair of laryngopharyngeal laceration and laryngeal laceration.

All the wounds were thoroughly irrigated with saline. The right laryngopharyngeal laceration was repaired with 4-0 Vicryl (Ethicon, Somerville, NJ) in two-layer closure. The fractured pieces of the anterior laryngeal cartilage were repaired with 4-0 Prolene suture (Surgi-pro; US Surgical, Norwalk, CT) (Figure 5). Cartilage takes approximately 3-5 months to heal. With polydioxanone (PDS) II suture (Ethicon, Somerville, NJ), wound support is only 60 days, thus we used nonabsorbable Prolene for a long duration of wound support to stabilize the thyroid cartilage. Laryngeal plating was not used to repair the laryngeal fracture due to wound contamination from an unvaccinated dog bite and the presence of multiple small fracture components. Multiple small dog bite skin injuries in the anterior neck and a left ear laceration were also repaired primarily with 4-0 Prolene. Laryngoscopy was completed at the end of the case to confirm proper reduction of the laryngeal cartilage fracture. The patient was brought to the emergency room in an unconscious state and a tracheostomy and percutaneous endoscopic gastrostomy (PEG) tube were completed.

The patient received a total of 1300 units of human rabies immunoglobulin (target dose of 20 units/kg) at the laceration sites at the time of surgery. The rabies human diploid cell vaccine (rabies vaccine), 1 ml intramuscular, was given on days #0, #3, #7 and #14. This patient was an immunocompetent patient; therefore, the fifth dose was not necessary. With extensive soft tissue and cartilaginous involvement, he was placed on 10 days of intravenous ampicillin-sulbactam for empiric coverage of cellulitis secondary to a dog bite, along with five days of ciprofloxacin for prevention of pseudomonas infection as recommended by the infectious disease physician. The patient stayed in the hospital for a total of 11 days postoperatively. He experienced transient dysphagia, but this resolved, and he recovered significantly during this period. On the 10th postoperative day, flexible laryngoscopy showed mobility of both vocal cords and his tracheostomy tube was decannulated successfully. The PEG tube was removed on the 15th postoperative day with no complications. No subglottic or supraglottic stenosis were noted two months postoperatively.

## Discussion

Dog bites are the leading cause of animal bites in humans and most often occur in the pediatric age group [2]. The majority of dog bites are superficial and close primarily [5]. Surgical management is only necessary for deep wounds. In contrast to old literature, the current guidelines recommend thorough irrigation with saline and hydrogen peroxide and immediate repair of the deep dog bite wounds to prevent cosmetic defects without increasing the risk of infection [6].

Since every deep bite wound is unique, different surgical techniques are used to manage each injury, including local flaps and free tissue transfer flaps. Laryngeal cartilage injuries are divided into five groups according to the Schaefer Classification System [7]. Groups 2-5 require surgical intervention in the operative room. This case involved a large right pharyngeal laceration at the level of the superior cornu of the thyroid cartilage along with an anterior thyroid cartilage fracture. Typically, adaptation plate fixation (APF) is used to repair the fracture of the laryngeal skeleton [8]. In this case, we opted for Prolene due to wound contamination from the unvaccinated dog bite and the presence of multiple small fracture fragments.

Deep dog bites are highly contaminated wounds with the most common bacteria including Streptococcus, Staphylococcus, Pasteurella, and other anaerobes [9]. The recommended treatment for optimal coverage includes a seven to 14-day course of amoxicillin-clavulanate [10]. Because the laryngeal cartilage fracture was contaminated, the patient received ampicillin-sulbactam for 10 days along with ciprofloxacin 500 mg twice daily for five days to prevent pseudomonas infection.

To date, there is no available literature describing pharyngeal laceration with thyroid cartilage fracture due to unvaccinated dog bite. We report the successful treatment of a severe dog bite injury to potentially guide future management of similar dog bite-related injuries.

## Conclusions

Dog bites are the most common animal bites in humans. The majority of injuries are superficial and require minimal medical intervention. Less frequently, dog bite injuries involving the airway, major blood vessels, and extensive tissue loss can be very serious. We report a case of successfully managing an extensive pharyngeal laceration and extensive laryngeal cartilage fracture with primary closure, without the use of laryngeal plating. This information will help the future management of similar cases.
